# Are Midlife Australians Ready to Implement Lifestyle Medicine to Improve Their Health and Wellbeing? A Cross-Sectional Online Survey

**DOI:** 10.1177/15598276261484859

**Published:** 2026-09-21

**Authors:** Jennifer Black, Sam Manger, Karen Carlisle

**Affiliations:** 1 College of Medicine and Dentistry, James Cook University, Douglas, QLD, Australia (JB, SM, KC)

**Keywords:** lifestyle medicine, midlife, acceptability, prevention, primary care, health coaching

## Abstract

This study assessed awareness, acceptability, and readiness to use Lifestyle Medicine (LM) among Australians in midlife (40-65 years). A cross-sectional online survey of Australian adults aged 40-65 years (n = 357) measured awareness and understanding of LM, willingness to use LM for prevention and after diagnosis, barriers to engagement, preferences for support, and readiness to discuss lifestyle change with a health professional. The survey included closed-response items and five open-ended questions. Awareness of the term LM was moderate (56%) and understanding was uneven, with many definitions emphasizing nutrition and exercise. LM was more acceptable for prevention (89.6% agreement) than after a new diagnosis (77%). Most participants supported discussing LM during routine health checks (75%) and valued personalized advice (85%). Readiness to discuss lifestyle change with a health professional was positive for 61%, while 31% were unsure. Qualitative findings highlighted the importance of timing, trust in clinicians, practical constraints, and ongoing support. Overall, midlife Australians reported strong acceptability of LM, particularly for prevention, but variable awareness and common practical barriers indicate a gap between endorsement and intended action. The findings support further evaluation of brief, personalized LM discussions and scalable follow-up pathways in routine care to determine whether these approaches can improve engagement and help close the knowing-to-doing gap.

“Participants most commonly used GPs or other health professionals (83%) and scientific research (77%) as sources of health information.”

## Introduction

Chronic disease remains the leading contributor to morbidity and premature mortality in Australia, creating growing demands on primary care and the broader health system.^1^ Midlife (40-65 years) represents a particularly important window for prevention as health risks and early chronic disease commonly emerge during this life stage, yet trajectories remain modifiable through targeted behavior change.^2^

Lifestyle Medicine (LM) is an evidence-informed approach that emphasizes therapeutic lifestyle behaviors and the environments that support them. The field typically focuses on six interrelated domains: healthy eating patterns, regular physical activity, restorative sleep, stress management, avoidance of risky substances and positive social connection and purpose.^3,4^ These pillars align with contemporary models of person-centered, preventive care and with synergistic approaches such as social prescribing and community-based health promotion.

Although lifestyle change is effective in preventing and helping manage many common chronic conditions, the integration of structured LM approaches into routine clinical care remains limited.^5,6^ In practice, lifestyle advice is often delivered briefly and variably, without consistent follow-up or behavior change support.^7^

Understanding acceptability is critical for implementation. Acceptability refers to the extent to which individuals perceive an intervention or approach as appropriate, based on anticipated or experienced benefits, burden, ethicality, and fit with personal values and circumstances.^8^ In the context of LM, acceptability is likely shaped by timing, including teachable moments, as well as by positive experiences, support, confidence, self-efficacy and barriers or resistance to change. Behavior change theory further suggests that intention alone is insufficient for sustained action. The Theory of Planned Behavior highlights the roles of attitudes, subjective norms, and perceived behavioral control in shaping intention and behavior.^9^ The Transtheoretical Model emphasizes readiness and stage-specific processes of change, suggesting that midlife adults may be positioned at different points along a continuum from precontemplation to maintenance.^10^ The Health Belief Model similarly suggests that perceived susceptibility, severity, benefits, and barriers influence engagement in preventive behaviors.^11^

A consistent theme in lifestyle interventions is the gap between “knowing” and “doing.” Middle-aged Australians describe a need for practical support, confidence, and accountability to translate health knowledge into sustained action.^12^ Evidence also indicates that delivery mode affects acceptability and engagement, with preferences often favoring trusted health care professionals and personalized approaches over generic messaging.^13,14^

### Study Aims

Despite increasing attention to LM in Australia, limited research has examined how midlife adults understand LM as a distinct concept, whether they perceive it as relevant to their current life stage, and how acceptable it is in different contexts. This study addresses this gap by investigating awareness and understanding of LM, alongside its acceptability and perceived relevance among Australians aged 40-65 years. It further examines whether acceptability differs when LM is framed as a preventive approach compared with use following a new diagnosis and explores factors influencing readiness to engage, including perceived barriers, support needs and preferences for delivery. Given the growing burden of chronic disease and the importance of midlife as a window of intervention, these insights have practical implications for improving how LM is communicated, introduced and supported in practice.

**Figure 1.**
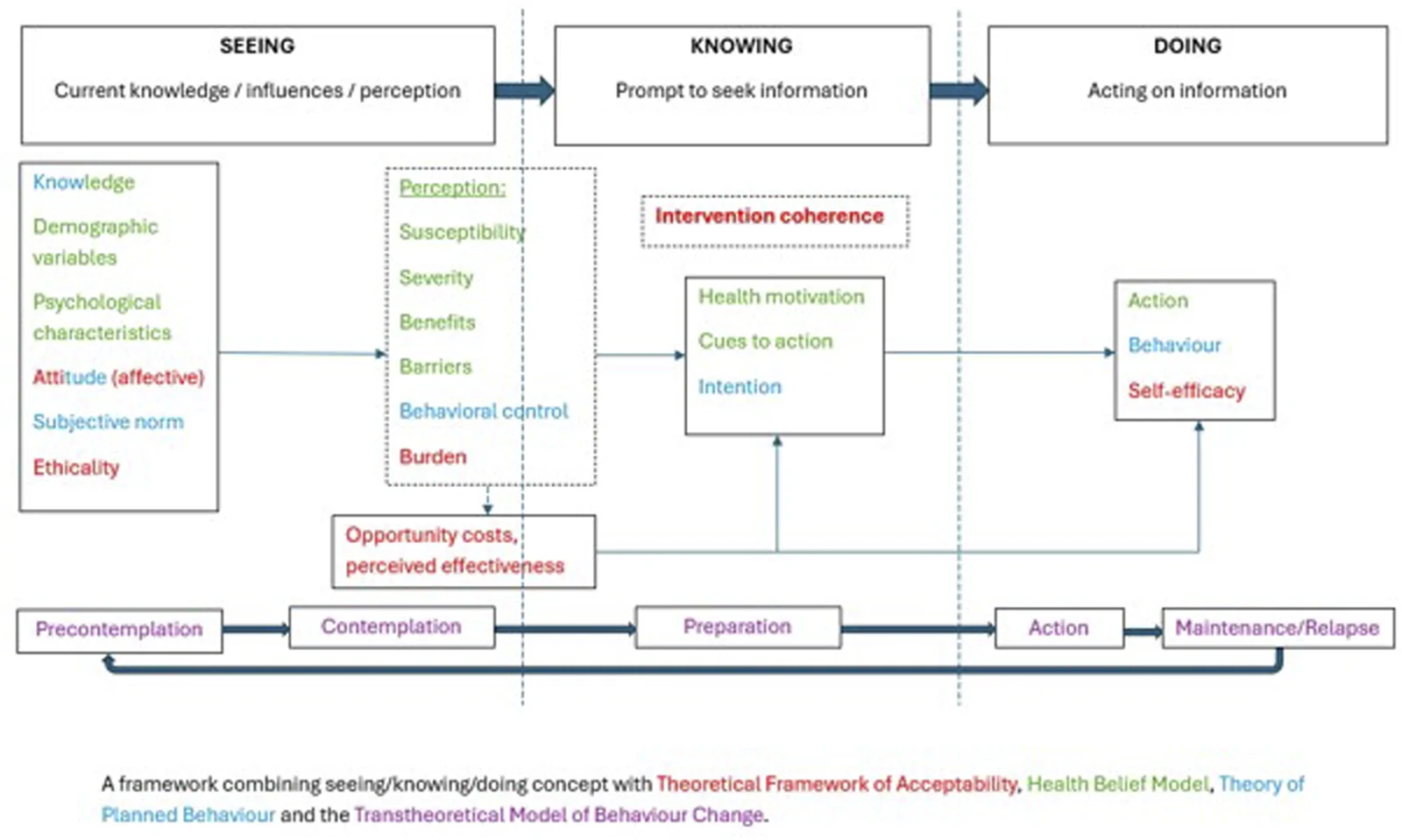
The Seeing-Knowing-Doing (SKD) Framework integrated with the theoretical framework of acceptability, health belief model, theory of planned behavior, and transtheoretical model, illustrating the synergy of behavior change theories and the process of health behavior transformation.

To support this investigation, the study used an online survey informed by Sterling’s Seeing–Knowing–Doing (SKD) framework^15^ (Figure 1), which draws together multiple behavior change theories into a visual model of how individuals move from perception and knowledge to reflection and behavioral implementation. Guided by this framework, the survey assessed awareness and understanding of LM, prompted reflection through brief education, and explored barriers, support needs, and readiness to act.

## Methods

### Study Design

A cross-sectional online survey was used to capture quantitative and qualitative information about awareness, acceptability and readiness to engage with LM among Australians in midlife.^16,17^ An online format, using convenience sampling, was selected to enable broad geographic reach, support anonymous participation, and allow inclusion of both structured responses and brief written reflections. This manuscript follows the CHERRIES (the Checklist for Reporting Results of Internet E-Surveys) guidelines.^18^ The study received ethical approval from James Cook’s Human Research Ethics Committee prior to data collection (Approval No. H9667).

The survey was administered using Qualtrics (Qualtrics, Provo, UT). Participants accessed a web link that opened a participant information page (providing survey length, advice that no personal data would be stored, that anonymized data may be used in future reporting and investigator and study purpose) followed by an electronic informed consent declaration. Only participants who provided consent could proceed to the survey items. Data were stored in password-protected systems consistent with institutional governance.

### Participants and Recruitment

Eligible participants were Australian adults aged 40 to 65 years. Recruitment was via advertisement posts on community social media platforms (Facebook and LinkedIn), using participant recruitment companies (StepUp for Ageing and Join Us) and by using the snowball effect. The participant recruitment companies are engagement services connecting individuals (who are willing to donate their time) with researchers. As these services connect researchers with individuals who have already expressed an interest in participating in research, this recruitment strategy was expected to increase the likelihood of enrolling participants who were more health-conscious, research-literate, or interested in aging and prevention than the broader midlife population. This potential selection bias was considered when interpreting levels of LM awareness, acceptability, and readiness. Participation was voluntary, non-incentivized, with no identifying information required within the survey itself. Given that the study examined perceptions and readiness rather than clinical outcomes, no a priori sample size calculation was used. The survey was conducted from 22 February 2025 to 1 April 2025.

### Survey Development and Measures

The open survey comprised 34 items over 12 pages (8 question pages, 1 video page and 3 information pages - survey questions listed in Table S1). Most items used closed-response options to support descriptive analysis, with five open-ended prompts to capture additional detail and to contextualize quantitative responses, and one question used adaptive questioning.^19^ Completeness was supported through mandatory questions, as participants were unable to move to the next page until a response was given. For some personal questions a “prefer not to say” option or the ability to leave the response blank was available. Due to the nature and flow of the survey, the back button was disabled. A unique response ID, assigned via cookies, was used to identify individual visitors and prevent duplicate entries during the survey period. The survey was intentionally organized into three sequential parts aligned with a Seeing-Knowing-Doing flow.

In Part 1 (Seeing), participants were asked about their awareness of the term “Lifestyle Medicine” and what it meant to them. This section also included items assessing perceptions of the relationship between lifestyle behaviors and chronic disease and general interest in lifestyle-based approaches.

In Part 2 (Knowing), participants viewed a brief informational video (video link provided in Table S1) introducing LM, its relevance to midlife and a short case example demonstrating lifestyle change in the context of chronic disease. Immediately following the video, participants were asked whether the content prompted reflection and to briefly describe what stood out to them.

In Part 3 (Doing), participants responded to items about willingness to apply LM to improve health and wellbeing, including willingness in prevention vs after diagnosis framing. Participants also reported perceived barriers to engaging in LM behaviors, preferred sources of health information, and preferences for support (e.g., learning from a general practitioner or health professional, scientific research, family and friends, or media). Additional items assessed the perceived value of personalized lifestyle advice and readiness to discuss lifestyle change with a health professional.

Open-ended responses were designed to be brief, typically one to three sentences. The analysis approach was therefore designed for brief survey text: rapid coding, content mapping to LM pillars, and inductive thematic synthesis with representative quotes. The survey was pretested for usability and technical functionality.

### Data Analysis

Quantitative analysis, using Qualtrics (version: March 2025) and Microsoft Excel (version 365), combined descriptive and inferential statistics. Incomplete survey responses were reviewed to assess the availability of demographic information and patterns of attrition. Incomplete responses were excluded from the primary analysis because they did not contain sufficient outcome data for inclusion. Frequencies, percentages, means, and standard deviations were used to summarize participant characteristics and key survey responses. As the main outcomes were ordinal Likert items, descriptive means were used for summary purposes, while non-parametric or categorical tests were prioritized for inference where appropriate.

Pearson’s chi-square tests examined associations between readiness to discuss lifestyle change and selected demographic variables (awareness of LM, chronic disease status, gender, age bracket and education). A paired Wilcoxon signed-rank test compared willingness to use LM for prevention vs after a new chronic disease diagnosis. To provide an overall measure of receptiveness to LM, a seven-item acceptability scale was created from core Likert items relating to prevention, post-diagnosis use, health-check discussions, personalized advice, professional support, family or friend support and readiness to discuss change. Internal consistency was acceptable (Cronbach’s α = 0.77). Independent-samples t-tests and one-way ANOVA were used to compare mean acceptability scores across selected subgroups. Spearman correlations examined relationships between acceptability, readiness, motivation after learning about LM, and valuing health information. Binary logistic regression was used to explore whether readiness to discuss change was associated with awareness, chronic disease history, age, gender, and education. Exploratory principal component analysis (PCA) was used to examine whether the acceptability items reflected a broader LM receptiveness construct.

Qualitative analysis combined content analysis and inductive thematic analysis. First, participants’ definitions of LM were examined using word-frequency approaches and content mapping to the six LM pillars to assess breadth of understanding. Inductive thematic analysis was then applied to the open-ended responses relating to motivation after the video, personalized lifestyle change, readiness to discuss change, and additional comments. This process was adapted from Naeem et al^20^ for short survey responses rather than full interview transcripts. Responses following the video were grouped into themes describing how the video influenced reflection and motivation, while responses relating to barriers, support needs, and readiness were synthesized into higher-level themes with illustrative quotes. Coding focused on common patterns relevant to acceptability, including perceived benefits, burden, opportunity costs, support, confidence, and trust.

## Results

### Participant Characteristics

Participants who started the survey included 485 Australian adults between the ages of 40-65 years. Of these, 128 participants failed to complete the survey resulting in a sample size of 357 (completeness rate: 74%). Incomplete responses were reviewed to examine survey progress and the availability of demographic information. However, many participants withdrew before providing sufficient demographic and outcome data, preventing a reliable comparison between completers and non-completers. The possibility that those who discontinued differed in health interest, time availability, digital engagement, or receptiveness to LM therefore cannot be excluded. Participants were predominantly female (78%) and tertiary educated (78%), and 72% reported current employment. The predominance of women and tertiary-educated participants was consistent with the anticipated selection effects of recruitment through research-engagement platforms, social media, and participant referral. The sample profile should therefore be considered when interpreting the comparatively high levels of interest in health information and receptiveness to LM reported below. The largest age bracket was 60-65 years (39%), and almost half reported ≥1 diagnosed chronic disease (49%). Due to very low representation of non-binary/non-conforming participants (*n* = 1), gender-stratified analyses were limited to male and female respondents (Table 1).

**Table 1.** Participant Demographics and Characteristics.

Demographic Characteristics	Survey Participants *N* = 357 Count %	
Gender		
Male	79	22
Female	277	78
Non-binary/Non-conforming	1	<1
Age bracket		
40-44	34	10
45-49	52	15
50-54	61	17
55-59	72	20
60-65	138	39
Highest education^a^		
Did not finish school	10	3
High school	67	19
Undergrad	111	31
Postgrad	167	47
Diagnosed with one or more chronic disease		
Yes	174	49
No	170	48
Not sure	13	4

a Missing responses excluded *n *= 2

### SEEING: Awareness, Understanding, and Current Health Context

Just over half of participants (56%, *n* = 200) reported awareness of the term “Lifestyle Medicine” (LM), with similar awareness by gender (males 58%, females 56%; Table 2). Open-text definitions indicated variable awareness. When responses were mapped to LM pillars, participants most frequently referenced nutrition (*n* = 110) and physical activity (*n* = 108), followed by social connection (*n* = 36), sleep (*n* = 33), stress/relaxation (*n* = 31), and substance avoidance (*n* = 21; Table S2). Although participants were not explicitly asked to list the six pillars, 109/357 responses referenced ≥1 pillar; only two participants named all six pillars, and the most common number of pillars referenced was two (*n* = 42), typically nutrition and exercise (Table S2).

**Table 2.** Key Awareness, Acceptability, and Readiness Outcomes.

Outcome	Yes	No	Strongly disagree	Disagree	Neither	Agree	Strongly agree	Agree/Strongly agree	Mean (1-5)
N = 357 *N* (%)									
SEEING – Awareness, understanding and current health context									
Awareness of LM term (Q2)	200 (56)	157 (44)	​	​	​	​	​	​	​
Male awareness of LM term (Q2)	46 (58)	33 (42)	​	​	​	​	​	​	​
Female awareness of LM term (Q2)	154 (56)	123 (44)	​	​	​	​	​	​	​
Currently in good overall health (Q4)	​	​	11 (3)	46 (13)	50 (14)	191 (54)	59 (17)	250 (70)	3.68
Learning about health is important (Q8)	​	​	1 (0)	0 (0)	11 (3)	117 (33)	228 (64)	345 (97)	4.60
KNOWING/DOING – Learning and useability of LM after watching educational video									
LM educational video motivated reflection on lifestyle (Q10)	​	​	2 (1)	11 (3)	71 (20)	176 (49)	97 (27)	273 (77)	3.99
Use LM for prevention (Q12)	​	​	1 (0)	4 (1)	32 (9)	147 (41)	173 (49)	320 (90)	4.36
Use LM after diagnosis (Q13)	​	​	8 (2)	20 (6)	55 (16)	118 (33)	156 (43)	274 (77)	4.10
Discuss LM at health checkup/screening (Q15)	​	​	4 (1)	27 (8)	59 (16)	133 (37)	134 (38)	267 (75)	4.03
Personalized lifestyle advice would encourage change (Q17)	​	​	1 (0)	4 (1)	49 (14)	161 (45)	142 (40)	303 (85)	4.23
Would like professional guidance/support (Q19)	​	​	5 (1)	33 (9)	91 (26)	124 (35)	104 (29)	228 (64)	3.81
Would like peer support group (Q20)	​	​	16 (5)	55 (15)	137 (38)	95 (27)	54 (15)	149 (42)	3.32
Have family/friends support (Q21)	​	​	13 (4)	21 (6)	60 (17)	168 (47)	95 (27)	263 (74)	3.87
Ready to discuss lifestyle change with professional (Q22)	​	​	5 (1)	24 (7)	109 (31)	141 (40)	78 (22)	219 (61)	3.74

Qualitative responses to the question *“To me, lifestyle medicine means… (please enter what you believe the term means),”* demonstrated substantial variation in understanding. Many descriptions focused on lifestyle factors broadly and on nutrition and exercise specifically, whereas fewer spontaneously referenced sleep, stress management, substance use or social connection as therapeutic targets. Some participants provided holistic definitions that aligned closely with LM principles, while others who had not previously heard the term still inferred an evidence-based behavioral approach. However, a minority associated LM with complementary or alternative medicine, supplements, or non-pharmacological therapies in ways suggesting that the terminology itself can mislead (Table 3). Taken together, these findings indicate that the seeing phase was only partially achieved: respondents often recognized the principles of LM without sharing a clear understanding of the label.

**Table 3.** Examples of Open-Ended Responses by Self-Reported Awareness of the Term Lifestyle Medicine and Level of Understanding.

Participants aware of the term LM with a good understanding; quotes	Participants aware of the term LM but with a lesser understanding; quotes
“Holistic” “Focus on prevention” “Lifestyle improvements” “Evidence based practices” “Behavioural changes” “Prevent, detect and minimise health concerns” “Actions… which are protective, preventative or remedial of diseases” “Whole person approach” “An aim to maximise positive life-years” “Person centred” “Healthy habits” “It acknowledges that health is not a condition but an expression of how we live our lives”	“Drugs that treat conditions related to someone’s lifestyle” “Medication you take for the rest of your life daily” “Medicine suited to the way we live and how we want to live” “Medicine to improve your enjoyment of life” “Medicine/medical way of living… a medical diet with supplements” “Medicines that may complement standard medications” “Alternative” “Complimentary” “Acupuncture” “Homeopathic” “Medicines such as ozempic which improve lifestyle for overweight or obese people” “Medicines to slow down ageing” “Unregulated chemicals” “Non-regulated alternative medicines”

Participants unaware of the term LM but with a good understanding; quotes	Participants unaware of the term LM and with a lesser understanding; quotes
“Positive changes to one’s lifestyle [*sic*]” “Using lifestyle changes… to prevent (and treat) chronic conditions” “Choices that you make in how you live your life and how they impact your health” “Making lifestyle decisions based on proven positive health benefits” “Work/life balance” “I haven’t heard this term before but I assume it means things like ‘prescribing' exercise, good diet, etc. lifestyle factors that can improve health, instead of medicine (e.g., low GI and exercise to manage diabetes).”	“Alternative medicine” “Non-western medicine” “Make-believe medicine” “Practitioners won’t have studied a medical degree” “Things one does or takes to improve health which are not prescribed or suggested by a doctor” “Medicine that purports to improve or manage perceived deficits in ideal lifestyle” “Taking medicine/drugs to improve one’s lifestyle” “Cannabis or similar drugs that would not be prescribed by a doctor” “I fear this is another term for wellness… ‘snake-oil’. As someone with cerebral palsy I would prefer regenerative medicine and cure to be the focus of lifestyle medicine.”

Overall, 70% of participants agreed or strongly agreed that they were currently in good health (Table 2). Self-perceived health varied by education level, with a significant association between higher education and more positive health ratings (χ^2^ *P* = .000357; Table S3a). Participants also differed in how much they had considered what aging may mean for their health, with more respondents strongly agreeing in the older category (Table S3b). These patterns suggest that reflection on health risk was uneven across the cohort before the LM material was introduced.

Learning about health was highly valued, with 97% agreeing or strongly agreeing that it was important (Table 2). Participants most commonly used GPs or other health professionals (83%) and scientific research (77%) as sources of health information. Social media was used by 44%, with higher use among participants in their 40s and 50s than those in their 60s (Table 4). This suggests that the seeing phase was already grounded in formal and trusted information sources for many participants.

**Table 4.** Sources of Information Used to Learn About Ways to Keep Healthy by Age Group and Gender (Q9).

Information Source	N = 357 Count (%)				​	​
	All	40s^a^	50s^b^	60s^c^	Males	Females
GP or health professional	296 (83)	71 (83)	110 (83)	115 (83)	64 (81)	231 (83)
Scientific research	273 (77)	67 (78)	109 (82)	97 (70)	54 (68)	218 (79)
Family/friends	174 (49)	46 (54)	64 (48)	64 (46)	45 (57)	129 (47)
News	172 (48)	35 (41)	61 (46)	76 (55)	41 (52)	131 (47)
Social media	158 (44)	41 (48)	63 (47)	54 (39)	31 (39)	126 (46)
Government notices	156 (44)	36 (42)	60 (45)	60 (44)	31 (39)	125 (45)
TV shows/documentaries	154 (43)	31 (36)	58 (44)	65 (47)	23 (29)	131 (47)
Other	52 (15)	13 (15)	21 (16)	18 (13)	10 (13)	42 (15)
None	2 (1)	1 (1)	0 (0)	1 (1)	2 (3)	0 (0.0)

^a^Total number in 40s group = 86.

^b^Total number in 50s group = 133.

^c^Total number in 60s group = 138.

*Notes*. Participants could select all options that applied.

### KNOWING: Learning Preferences, Intervention Response and Acceptability

After viewing the 5-minute LM educational video, 77% reported it motivated them to reflect on their lifestyle (Table 2). Thematic analysis identified five positive sub-themes: new awareness/motivation, behavior change intentions, reflections on midlife and aging, power of lifestyle change, and renewed commitment. Neutral or negative responses reflected existing familiarity with the content, difficulty implementing change, unfamiliarity with the term LM despite recognizing its components, and skepticism about aspects of the case study and scientific justification. These mixed reactions indicate that many participants reported reflecting on their lifestyle after viewing the brief educational content, although the study design cannot establish whether the video produced or sustained changes in motivation or behavior.

LM was viewed more favorably when framed as prevention than when framed as something to use after a new chronic disease diagnosis. For prevention, 320 of 357 participants (90%) agreed or strongly agreed that they would be interested in using LM activities, compared with 274 of 357 (77%) in the newly diagnosed scenario (Table 2). This within-person difference was statistically significant (Wilcoxon signed-rank W = 4745.5, *P* < .001; Table S4), showing stronger support for LM as a preventive approach than as a treatment-adjunct approach. The distribution of paired score differences is shown in Table S4a.

Nearly one-third of participants (30%) selected “None” when asked about barriers to engaging in LM activities. Of those respondents who reported barriers, the most frequently identified challenges included lack of motivation or energy (35%), constraints related to job or work hours (20%), issues concerning location, travel, or cost (19%), insufficient knowledge regarding appropriate actions or methods (17%), limited available time (17%), and compromised physical or mental health (17%; Table 5). Barriers differed by age, with respondents in their 40s more likely to report work, time, and family constraints, whereas respondents in their 60s were more likely to report no barriers (Table 5). These findings suggest that the shift from knowing to doing was limited more by practical burden than by resistance to the ideas themselves.

**Table 5.** Reported Barriers to Engaging in Lifestyle Medicine Activities by Age Group.

Barriers	*N* = 357 Count (%)			
	All	40s^a^	50s^b^	60s^c^
Motivation/energy	126 (35)	29 (34)	55 (41)	42 (30)
None	106 (30)	16 (19)	34 (26)	56 (41)
Job (sedentary work/work hours)	71 (20)	26 (30)	29 (22)	16 (12)
Location/travel/cost	67 (19)	19 (22)	26 (20)	22 (16)
Knowledge (of what to do/how to do it)	62 (17)	19 (22)	24 (18)	19 (14)
No time	59 (17)	24 (28)	29 (22)	6 (4)
Poor physical or mental health	59 (17)	8 (9)	28 (21)	23 (17)
Family commitments (including childcare)	54 (15)	26 (30)	18 (14)	10 (7)
No support	47 (13)	9 (10)	16 (12)	22 (16)
Outside conditions (e.g., weather)	42 (12)	8 (9)	15 (11)	19 (14)
Other	35 (10)	10 (12)	14 (11)	11 (8)
Facilities	33 (9)	8 (9)	10 (8)	15 (11)
Fear of injury	31 (9)	5 (6)	15 (11)	11 (8)
Safety (e.g., walking alone)	27 (8)	4 (5)	11 (8)	12 (9)

^a^Total number in 40s group = 86.

^b^Total number in 50s group = 133.

^c^Total number in 60s group = 138.

*Notes*. Participants could select all options that applied.

Three-quarters (75%) agreed or strongly agreed they would like a GP or health professional to discuss LM activities during a health check or screening appointment and 85% agreed/strongly agreed that learning about lifestyle changes “appropriate to me” would encourage change (Table 2). When combined into the seven-item acceptability scale, overall LM receptiveness was high (mean approximately 4.02 on a 5-point scale) and internally consistent (Cronbach’s α = 0.77; Table 6; calculation details in Table S5). PCA suggested that these items reflected a broader underlying LM receptiveness pattern (Table S6), with details of eigenvalues and explained variance shown in Table S6a and item loadings shown in Table S6b.

**Table 6.** Seven-Item Acceptability Scale: Item Summaries and Internal Consistency.

Question number	Scale item	Mean Score (SD)	Agree/Strongly agree *N* (%)
		*N* = 357	
Q12	Interested in using LM for prevention	4.36 (0.72)	320 (90)
Q13	Likely to use LM after new diagnosis	4.1 (1.0)	274 (77)
Q15	Would like LM discussed at health check	4.03 (0.97)	267 (75)
Q17	Personalized advice would encourage change	4.23 (0.74)	303 (85)
Q19	Would like professional guidance/support	3.81 (1.0)	228 (64)
Q21	Have family/friends support	3.87 (0.99)	263 (74)
Q22	Ready to discuss lifestyle change with professional	3.74 (0.92)	219 (61)

*Notes.* Overall scale mean = 4.02; SD = 0.59; Cronbach alpha = 0.768.

### DOING: Support needs and readiness to engage

Participants most commonly identified family and friends as likely sources of support for future lifestyle change (74%), followed by health professional support (64%) and peer support (42%; Table 2). This suggests that participants preferred support that combined existing personal relationships with professional guidance, rather than relying on peer-group formats alone.

Most participants indicated readiness to engage with professional support for lifestyle change: 61% expressed a positive response to discussing lifestyle change with a health professional, 31% were unsure, and 8% disagreed (Table 2). Readiness, learning about health and motivation after watching the LM video were all strongly associated with the overall acceptability scale (Spearman ρ = 0.743, *P* < .001; ρ = 0.349, *P* < .001; ρ = 0.514, *P* < .001, Table S7). Awareness of the term LM itself showed only a marginally significant association with acceptability (ρ = 0.102, *P* = .055; Table S7), indicating that awareness of the LM term alone was not strongly associated with reported readiness in this sample.

Chi-square analyses showed that readiness to discuss lifestyle change was significantly associated with chronic disease history (χ^2^ = 7.65, df = 2, *P* = .0219; Cramer’s V = 0.146; Table S8), whereas associations with awareness of LM, gender, age bracket, and education did not reach conventional significance (Table S8). The corresponding cross-tabulations are shown in Table S8a. Mean acceptability was slightly higher among respondents with chronic disease than among those without (4.10 vs 3.95; Table S9; Welch’s t = 2.245, df = 336.939, *P* = .0254; Cohen’s d = 0.24; Table S9a). Acceptability also differed modestly across age groups overall, with the lowest mean in the 40-44 age group (3.82) and the highest in the 45-49 age group (4.23; Table S9b); this overall difference was statistically significant on one-way ANOVA (F = 3.29, df = 4, 352, *P* = .0114; Table S9c), with post-hoc comparisons shown in Table S9d.

Multivariable logistic regression was then used to examine which factors were independently associated with positive readiness to discuss lifestyle change. Greater readiness was associated with being aged 45-49 years compared with 40-44 years (OR = 3.42, 95% CI = 1.31-8.93, *P* = .0121) and with having undergraduate education compared with high school education (OR = 2.10, 95% CI = 1.08-4.07, *P* = .0284; Table S13). Chronic disease history and awareness of the LM term both approached significance after adjustment (OR = 1.56, 95% CI = 0.99-2.48, *P* = .0577; and OR = 1.57, 95% CI = 1.00-2.48, *P* = .0517, respectively; Table S10). These adjusted findings indicate that reported readiness varied across selected age and education categories, although the cross-sectional design does not establish why these differences occurred.

Qualitative responses suggested that uncertainty in making lifestyle changes was often related to competing priorities, prior experiences of brief or non-specific advice, or uncertainty about what support would involve (Table 7). Among those who disagreed about discussing lifestyle changes with a health professional, the main reasons clustered into six themes: perceived self-sufficiency, practical barriers, lack of trust in professionals, emotional or psychological barriers, skepticism or rejection of the concept, and cautious expertise (e.g., prior knowledge or professional background). These themes suggest that how LM conversations are introduced, framed, and supported over time may be important for engagement.

**Table 7.** Thematic Summary of Reasons for Not Wanting to Discuss Lifestyle Change With a Health Professional.

Theme	Summary	Illustrative Quotes
1. Perceived Self-Sufficiency	Participants felt confident in their health knowledge or behaviors and did not perceive a need for professional guidance.	“I feel like I know what I have to do.” “I already lead a healthy lifestyle.” “I live a healthy life as it is.” “I’m already doing everything you identified in your video.” “Fairly happy with my health and rhythms at this time.”
2. Practical Barriers	Time constraints, cost, and limited health care access were key deterrents to engaging in conversations with professionals.	“Lack of time.” “Cost of seeking medical support and lack of facilities/unable to get into a Dr regularly.” “Just not at this time, FT work and family.” “Making time to meet with someone would be difficult when I already have a full schedule.” “Why would you just start having conversations with your GP about this if you don’t need to?…”
3. Lack of Trust in Professionals	Distrust in the health care system or dissatisfaction with past experiences, especially with GPs, discouraged participants.	“Gps are not trained nor have the time interest or education to facilitate this.” “I don’t think GP’s actually care to have these type of conversations.” “I have tried it before and haven’t managed to make a change. Also, definitely not a GP. It always feels like being chastised.” “Judgement, presumptive assumptions… being dismissed.” “I don’t have enough of a relationship with my GP for her to have a big influence.”
4. Emotional or Psychological Barriers	Mental health struggles, illness, or emotional overwhelm made lifestyle discussions feel unattainable or too confronting.	“Because I’m dealing with massive postnatal depression right now… completely impossible.” “Currently fighting severe illness.” “Scared. Frightened. Previous failed attempts.” “Not sure, perhaps not wanting to discuss the hard reality.” “Don’t feel a pressing need in terms of my current health.”
5. Skepticism or Rejection	Some participants rejected the medicalization of lifestyle change or questioned the legitimacy of the approach.	“I think it’s a load of bullshit.” “This is trying too hard to medicalise what should be healthy living strategies.” “I do not enjoy this social learning model.” “Don’t like being told what to do, or to change at someone else’s pace.” “I would never see a life coach anyway as it's largely a scam profession imo.”
6. Cautious Expertise	Participants with prior experience or health knowledge expressed caution about conflicting advice or over-medicalization	“Sorry this may be unfair… I have a high level of knowledge in this area… I follow my chosen path.” “I am already doing what is possible and have found much advice from GPs to be woeful.” “Several ambulance trips have fortified my resolve.” “Further damage can be done by following misinformed or misguided advice.” “I can see there are numerous interpretations of what is and isn’t healthy.”

## Discussion

This study examined awareness, attitudes, and readiness among midlife Australians to engage with LM to support health and prevent chronic disease. Using the SKD framework, the findings suggest that progress across these stages was uneven rather than absent. In the seeing phase, awareness of the term LM was only moderate and understanding was inconsistent. In the knowing phase, participants were highly open to learning and generally responded positively to brief education, especially when information felt relevant and trustworthy. In the doing phase, willingness was broadly positive, but action was constrained by motivation, time, cost, competing responsibilities, and uncertainty about how to turn knowledge into sustained behavior change. Overall, the findings point less to rejection of LM than to a gap between endorsement and implementation, aligning with the Theory of Planned Behavior’s emphasis on perceived behavioral control as a key influence on whether intention translates into action.

### Education - Awareness and Health Literacy

Participants frequently recognized the principles of LM without knowing the term or understanding its full scope, consistent with evidence that LM awareness remains limited among both the public and health professionals.^21^ Awareness of the label was only weakly associated with acceptability and readiness, suggesting that communication may need to prioritize clear explanations of relevant behaviors and benefits rather than relying on LM terminology alone. In SKD terms, seeing the concept was not the same as knowing it well enough to act on it confidently.

Brief education prompted reported reflection for many participants, but practical barriers remained. This is consistent with the intention-behavior gap described by the Health Belief Model and Theory of Planned Behavior whereby positive attitudes may not translate into action when perceived barriers, competing demands or low perceived behavioral control remain high.^9,11,22^

### Education - Personalization and Timing

Participants strongly endorsed personalized advice and discussion during routine care, indicating a preference for information that is applicable, credible, and tailored, consistent with previous research.^23,24^ Receptiveness may depend not only on general approval of LM, but also on whether participants could envisage a realistic and supported way of engaging with it. This is relevant to concerns about limited consultation time, uneven LM training, and cost barriers in primary care.^5,7^ Models such as health coaching and shared medical appointments warrant further evaluation as possible ways of extending personalized support beyond the standard GP visit.^6,14,25^

Receptiveness may also vary across midlife as work, family responsibilities, perceived health risks, and readiness change over time. Greater reflection on aging among older participants is consistent with the concept of “Emerging Elderhood.”^26^ LM communication and delivery may therefore need to account for differences in life-stage context.

Prevention-oriented framing was more acceptable than post-diagnosis framing, while chronic disease history was associated with greater readiness. Routine health checks, screening appointments, and a new diagnosis may therefore represent potential teachable moments in which brief LM conversations could be evaluated.^27–29^

Brief digital resources may complement personalized discussion, particularly when they are accessible, visual and evidence-based.^30,31^ Their reach and effects on sustained engagement require further evaluation.

### Support - Barriers to Change

Reported barriers were mainly motivational and structural rather than reflecting rejection of LM. This aligns with evidence concerning self-efficacy and the difficulties individuals experience in sustaining behavior change.^32,33^ The findings support the concept of a knowing-to-doing gap, in which awareness and positive intentions may be insufficient when time, energy, cost, competing responsibilities and limited perceived control create practical friction.

Strategies such as realistic goal setting, accountability, follow-up support, and assistance with confidence and pacing may be appropriate for future intervention studies.^34^ Their effectiveness in supporting sustained behavior change cannot be determined from the present findings.

Practical constraints appeared particularly relevant to younger midlife adults, who may experience competing work, family and caregiving demands.^13^ This is consistent with behavior change literature showing that engagement is influenced by perceived barriers, self-efficacy, social context and available support rather than demographic characteristics alone.^9,11,22^ The regression findings should therefore be interpreted as reflecting potentially overlapping demographic and contextual influences rather than isolated causal effects.^9,11^

### Support - The Role of Support

Participants expressed a preference for support involving trusted personal relationships and professional guidance. This is consistent with evidence that social connection, accountability, and encouragement can support behavior adoption and maintenance.^35,36^ Although peer support was less strongly endorsed, it may still be acceptable when organized around shared purpose and practical accountability.^37–39^

A layered model combining brief primary care initiation with health coaching, digital follow-up, and family or community support may therefore warrant evaluation for acceptability, feasibility, effectiveness and cost.

### Support - Readiness for Change

Although many participants reported readiness to discuss lifestyle change, uncertainty was linked to competing priorities, previous experiences of non-specific advice, emotional burden, and uncertainty about what support would involve. Responses to the educational video were similarly mixed. As there was no control condition or longitudinal follow-up, it cannot be concluded that the video caused or sustained changes in motivation. The findings instead support further evaluation of brief education combined with tailored discussion, practical options and ongoing support. This is consistent with evidence that prompts may encourage reflection but are more likely to support change when embedded within broader behavior change strategies.^40,41^

Overall, the findings indicate that many participants endorsed healthier living in principle while also reporting barriers to implementation. Future research should examine how the timing, framing, and delivery of LM support influence actual engagement and sustained behavior change.

### Strengths and Limitations

Strengths of this study include its focus on a specific but under-examined life stage, the use of a theoretically informed survey structure, and the integration of quantitative and qualitative data to capture not only attitudes toward LM but also the reasoning behind them. Use of the SKD framework provided a useful organizing lens for interpreting how awareness, learning, and readiness related to one another. The inclusion of a brief educational video also allowed exploration of how scalable, low-intensity information may prompt reflection among midlife adults.

Several limitations should be considered. Convenience sampling through social media, participant referral and health and aging research platforms may have attracted people who were more health-conscious, research-literate, or comfortable with online surveys than the wider midlife population. The predominance of women and tertiary-educated respondents may therefore have contributed to the high levels of interest in health information and receptiveness to LM, limiting generalizability.

Of the 485 participants who commenced the survey, 128 did not complete it. As insufficient information was available for a reliable comparison between completers and non-completers, it is unclear whether attrition introduced additional bias. Participants who were less interested, more time-constrained, or less comfortable with the online format may have been more likely to discontinue. Future online studies should collect essential demographic information early, monitor attrition by survey stage, and consider reducing survey length or burden to improve completion.

The findings were based on self-reported attitudes and intentions rather than observed behavior and may have been influenced by social desirability or short-term prompting from the educational video. The cross-sectional design also prevents causal conclusions; associations should therefore be interpreted as exploratory.

Finally, the open-ended responses provided useful context but lacked the depth of interviews or focus groups. Further qualitative and longitudinal research is needed to examine whether reported readiness translates into sustained engagement and behavior change.

## Future Research and Implementation Implications

Future research should purposively recruit men, culturally and linguistically diverse communities, people with lower educational or digital literacy and rural or remote populations to improve representativeness. Qualitative studies could further examine skepticism, mistrust, previous experiences of lifestyle advice, and the practical circumstances influencing readiness to engage.

Longitudinal and intervention studies are also needed to determine whether reported acceptability translates into participation and sustained behavior change. Specific strategies for evaluation include brief structured LM conversations delivered at potential teachable moments, such as routine health checks, screening appointments, and following a new diagnosis, with content tailored to individual readiness, priorities and perceived barriers. Studies should compare different delivery approaches, including conversations initiated by GPs, nurses, or allied health professionals and supported by appropriate written or digital resources.

Implementation research should also examine multidisciplinary referral pathways linking primary care with health coaching, dietetics, exercise physiology, psychology, social prescribing and community-based services. Models combining brief primary care initiation with follow-up coaching, digital support, and family or household involvement may be particularly relevant to the preferences identified in this study. Future evaluations should assess reach, acceptability, feasibility, equity, fidelity, cost, participant engagement, and longer-term behavioral and clinical outcomes. Such research would help determine which approaches are most appropriate for different stages of midlife and whether they can help close the knowing-to-doing gap.

## Conclusions

This study indicates that midlife Australians generally perceive LM as acceptable and relevant, particularly when it is framed as prevention and introduced through trusted professional relationships. However, awareness of the term LM and understanding of its full scope were variable, while motivational and practical barriers may interrupt progression from awareness and reflection to sustained action.

As these findings are cross-sectional and self-reported, they identify associations and participant preferences rather than demonstrating the effectiveness of particular approaches. The findings support further evaluation of brief, personalized LM discussions in routine care and of scalable follow-up options including coaching, digital resources, and community-based support, to determine whether these approaches can improve engagement and sustained behavior change, thereby helping to close the knowing-to-doing gap at an important life stage for prevention.

## Supplemental Material

Suppplemental Material - Are Midlife Australians Ready to Implement Lifestyle Medicine to Improve Their Health and Wellbeing? A Cross-Sectional Online Survey Suppplemental Material for Are Midlife Australians Ready to Implement Lifestyle Medicine to Improve Their Health and Wellbeing? A Cross-Sectional Online Survey by Jennifer Black, Sam Manger, Karen Carlisle in American Journal of Lifestyle Medicine

## Data Availability

The datasets generated and/or analysed during the current study are available from the corresponding author upon reasonable request.*
