# Supplementary material for: Are Midlife Australians Ready to Implement Lifestyle Medicine to Improve Their Health and Wellbeing? A Cross-Sectional Online Survey
Source: Am J Lifestyle Med. 2026 Sep 21:15598276261484859. Online ahead of print. doi: 10.1177/15598276261484859 (PMC13593945; doi:10.1177/15598276261484859)
Supplement: Supplementary file 1 [file sj-zip-1-ajl-10.1177_15598276261484859.zip › Table Supplementary 1.docx]

**Table S1.** Survey questions

| **No.** | **Question** | **Possible replies** |
| --- | --- | --- |
| **INTRO** | | |
| 1. | I consent to complete this survey | Yes/No |
| **SECTION ONE (SEEING)** | | |
| 2. | I am aware of the term lifestyle medicine | Yes/No |
| 3. | To me, lifestyle medicine means (please enter what you believe the term means)… | [short-text response] |
| 4. | I feel like I am currently in good overall health | - Strongly agree - Agree - Neither agree nor disagree - Disagree - Strongly disagree |
| 5. | I have considered what ageing may mean for my health | - Strongly agree - Agree - Neither agree nor disagree - Disagree - Strongly disagree |
| 6. | I currently look after my health and wellbeing in the following ways: (select all that apply) | - Regular exercise (2.5-5 hrs moderate physical activity and 1.25-2.5 hrs vigorous activity per week) - Healthy diet (x5 serves of veg and x2 serves of fruit every day most or all of the time) - Sleep well - Non-drinker / moderate alcohol (no more than 10 standard drinks a week and no more than 4 standard drinks on any one day) - Do not smoke - Healthy social connections - Managing stress levels - Taking time for myself - None - Other |
| 7. | I believe that the risk of chronic disease (e.g. diabetes, heart disease, obesity) is linked to poor lifestyle choices | - Strongly agree - Agree - Neither agree nor disagree - Disagree - Strongly disagree |
| 8. | Learning about my health is important to me | - Strongly agree - Agree - Neither agree nor disagree - Disagree - Strongly disagree |
| 9. | The sources of information I use to learn about ways to keep healthy are: (select all that apply) | - Family / friends - Social media - News - GP or other health professional - TV shows / documentaries - Scientific research - Government notices - None - Other |
| **SECTION TWO (KNOWING)** | | |
|  | Informational video | [Watch video](https://youtu.be/0yT25CNHh40) |
| **SECTION THREE (DOING)** | | |
| 10. | Learning more about lifestyle medicine has motivated me to consider the way I live | - Strongly agree - Agree - Neither agree nor disagree - Disagree - Strongly disagree |
| 11. | Please feel free to expand on the previous question if required | [short-text response] |
| 12. | I would be interested to use lifestyle medicine activities to help prevent a future chronic disease | - Strongly agree - Agree - Neither agree nor disagree - Disagree - Strongly disagree |
| 13. | I would more likely consider using lifestyle medicine activities if I was newly diagnosed with a chronic disease (in conjunction with other forms of treatment where necessary) | - Strongly agree - Agree - Neither agree nor disagree - Disagree - Strongly disagree |
| 14. | For me, engaging in lifestyle medicine activities would be difficult due to: (select all that apply) | - Job (sedentary work/work hours) - No time - Location/travel/cost - Family commitments (including childcare) - Poor physical or mental health - Motivation/energy - Knowledge (of what to do/how to do it) - No support - Outside conditions (e.g. weather) - Fear of injury - Safety (e.g. walking alone) - Facilities - None - Other... |
| 15. | I would like my GP/health professional to discuss lifestyle medicine activities that would be beneficial to me at an appointment such as a health checkup or screening event | - Strongly agree - Agree - Neither agree nor disagree - Disagree - Strongly disagree |
| 16. | If I were to receive more information about lifestyle medicine, I would prefer it to be via: (select all that apply) | - In person, one-on-one conversation (personalised in depth conversation) - In person, focus group (small, moderated discussion with peer-to-peer sharing) - In person, interactive (e.g. lecture or panel discussion with experts, events/fairs) - Digital, quick read (e.g. blogs, website, social media/email campaign, infographics) - Digital, more in-depth (e.g. articles, e-books) - Hardcopy, quick read (e.g. poster, flyer) - Hardcopy, more in depth (e.g. books) - Digital media with no engagement (e.g. TED-style talks, videos/documentaries, social   media posts, short animations, TV show, radio/podcast)   - Digital media with engagement (e.g. surveys/quizzes, online courses,   workshops/training/webinars, games/simulations, forums/online communities)   - Other… |
| 17. | Learning about positive lifestyle changes appropriate to me would encourage me to start making changes | - Strongly agree - Agree - Neither agree nor disagree - Disagree - Strongly disagree |
| 18. | Please feel free to expand on the previous question if required | [short-text response] |
| 19. | I would like a professional health coach/GP/health professional to help guide and support my lifestyle changes | - Strongly agree - Agree - Neither agree nor disagree - Disagree - Strongly disagree |
| 20. | When making lifestyle changes, I would like to join a support group of peers who were making similar changes | - Strongly agree - Agree - Neither agree nor disagree - Disagree - Strongly disagree |
| 21. | If I were to make lifestyle changes, I have people around me (  family/friends) who would  support the change | - Strongly agree - Agree - Neither agree nor disagree - Disagree - Strongly disagree |
| 22. | I feel ready to have a discussion with a health coach/GP/health professional about making  changes to my lifestyle to benefit my health | - Strongly agree - Agree - Neither agree nor disagree - Disagree - Strongly disagree |
| If Disagree or Strongly disagree selected in previous question, question No. 23 becomes available, if not question No. 23 is hidden | | |
| 23. | What is the main reason for not wanting a discussion? | [short-text response] |
| 24. | Please enter any other comments and consider what would encourage you to start making lifestyle changes? (if nothing, enter none) | [short-text response] |
| 25. | How would you describe your gender? | - Male - Female - Non-binary / Non-conforming - Prefer not to say - Other |
| 26. | Which age bracket are you in? | - 40-44 - 45-49 - 50-54 - 55-59 - 60-65 |
| 27. | How would you describe your ethnicity? (please leave blank if you would prefer not to say) | [short-text response] |
| 28. | Current family situation (please select all that apply) | - Single - De facto - Married - Divorced - Separated - Widowed - Child/children at home - Child/children have left home - Other adult (other than partner) living at home (e.g. parent) - Prefer not to say |
| 29. | What is your postcode? (4-digit code) | [short-text response] |
| 30. | What is your current employment status? (select all that apply) | - Full-time - Part-time - Business owner - Casual - Temporary - Contract - Student - At home carer - Retired - Not currently employed |
| 31. | What is your highest level of education? | - Did not finish school - High school - Undergrad - Postgrad |
| 32. | Have you ever been diagnosed with one or more chronic disease? | - Yes - No - Not sure |
| 33. | How did you find the link to this survey? | - Join Us - Step up for Research - Facebook - LinkedIn - Family/friend - Other |
| 34. | If other selected for last question, please explain... | [short-text response] |
