# Supplementary material for: Are Midlife Australians Ready to Implement Lifestyle Medicine to Improve Their Health and Wellbeing? A Cross-Sectional Online Survey
Source: Am J Lifestyle Med. 2026 Sep 21:15598276261484859. Online ahead of print. doi: 10.1177/15598276261484859 (PMC13593945; doi:10.1177/15598276261484859)
Supplement: Supplementary file 1 [file sj-zip-1-ajl-10.1177_15598276261484859.zip › Table Supplementary 10.docx]

**Table S10.** Multivariable logistic regression predicting positive readiness to discuss lifestyle change

| **Predictor** | **OR** | **95% CI lower** | **95% CI upper** | **p value** |
| --- | --- | --- | --- | --- |
| Chronic disease history: Not sure vs No | 0.476 | 0 | 2 | 0.2281 |
| Chronic disease history: Yes vs No | 1.564 | 1 | 2 | 0.0577 |
| Aware of LM term: Yes vs No | 1.572 | 1 | 2 | 0.0517 |
| Gender: Male vs Female | 1.048 | 1 | 2 | 0.8671 |
| Age 45-49 vs 40-44 | 3.42 | 1 | 9 | 0.0121 |
| Age 50-54 vs 40-44 | 1.18 | 0 | 3 | 0.7079 |
| Age 55-59 vs 40-44 | 1.133 | 0 | 3 | 0.7703 |
| Age 60-65 vs 40-44 | 1.562 | 1 | 3 | 0.2636 |
| Education: Did not finish school vs High school | 0.496 | 0 | 2 | 0.3234 |
| Education: Postgrad vs High school | 1.083 | 1 | 2 | 0.7953 |
| Education: Undergrad vs High school | 2.099 | 1 | 4 | 0.0284 |

*Note. Outcome coded as positive readiness (agree/strongly agree) vs not positive. Model n = 354 after excluding missing gender/education values.*
