# Supplementary material for: Are Midlife Australians Ready to Implement Lifestyle Medicine to Improve Their Health and Wellbeing? A Cross-Sectional Online Survey
Source: Am J Lifestyle Med. 2026 Sep 21:15598276261484859. Online ahead of print. doi: 10.1177/15598276261484859 (PMC13593945; doi:10.1177/15598276261484859)
Supplement: Supplementary file 1 [file sj-zip-1-ajl-10.1177_15598276261484859.zip › Table Supplementary 2.docx]

**Table S2.** Open-text definitions of Lifestyle Medicine mapped to the six LM pillars (relating to Q3)

| **LM pillar / No. of pillars** | **n mentioning pillar** | **% of total sample (357)** |
| --- | --- | --- |
| Nutrition | 110 | 31 |
| Physical activity / exercise | 108 | 30 |
| Social connection | 36 | 10 |
| Sleep | 33 | 9 |
| Stress/relaxation | 31 | 9 |
| Substance avoidance | 21 | 6 |
|  | | |
| At least one pillar | 109 | 31 |
| 1 Pillar | 13 | 4 |
| 2 Pillars | 42 | 12 |
| 3 Pillars | 23 | 6 |
| 4 Pillars | 19 | 5 |
| 5 Pillars | 10 | 3 |
| 6 Pillars | 2 | <1 |
| *Notes:* Counts are based on the coded qualitative responses supplied for the six pillar groupings. Responses could contribute to more than 1 pillar. | | |
