# Supplementary material for: Are Midlife Australians Ready to Implement Lifestyle Medicine to Improve Their Health and Wellbeing? A Cross-Sectional Online Survey
Source: Am J Lifestyle Med. 2026 Sep 21:15598276261484859. Online ahead of print. doi: 10.1177/15598276261484859 (PMC13593945; doi:10.1177/15598276261484859)
Supplement: Supplementary file 1 [file sj-zip-1-ajl-10.1177_15598276261484859.zip › Table Supplementary 3a.docx]

**Table S3a.** Chi-square test showing self-perceived health (Q4) by education level (Q31)

| **Analysis** | **Test** | **Chi-square** | **df** | **p value** |  |
| --- | --- | --- | --- | --- | --- |
| Self-perceived health by education level | Pearson chi-square | 29.288 | 12 | 0.003569 |  |
|  | **Q4 - I feel like I am currently in good overall health** | | | | |
| **Q31 -Education level** | **Strongly agree** | **Agree** | **Neither agree nor disagree** | **Disagree** | **Strongly disagree** |
| Did not finish school | 1 | 2 | 1 | 4 | 2 |
| High school | 9 | 33 | 13 | 9 | 3 |
| Undergrad | 15 | 56 | 19 | 18 | 3 |
| Postgrad | 34 | 98 | 17 | 15 | 3 |
