# Supplementary material for: Are Midlife Australians Ready to Implement Lifestyle Medicine to Improve Their Health and Wellbeing? A Cross-Sectional Online Survey
Source: Am J Lifestyle Med. 2026 Sep 21:15598276261484859. Online ahead of print. doi: 10.1177/15598276261484859 (PMC13593945; doi:10.1177/15598276261484859)
Supplement: Supplementary file 1 [file sj-zip-1-ajl-10.1177_15598276261484859.zip › Table Supplementary 3b.docx]

**Table S3b.** Chi-square test considered ageing implications (Q5) by age band (40s/50s/60s) (Q26)

| **Analysis** | **Test** | **Chi-square** | **df** | **p value** |  |
| --- | --- | --- | --- | --- | --- |
| Considered ageing implications by age band | Pearson chi-square | 16.332 | 6 | 0.01208 |  |
|  | **Q5 - I have considered what ageing may mean for my health** | | | | |
| **Q26 –**  **Age band** | **Strongly agree** | **Agree** | **Neither agree nor disagree** | **Disagree** | **Strongly disagree** |
| 40s | 27 | 55 | 2 | 2 | 0 |
| 50s | 62 | 65 | 5 | 1 | 0 |
| 60s | 79 | 56 | 2 | 1 | 0 |
