# Supplementary material for: Are Midlife Australians Ready to Implement Lifestyle Medicine to Improve Their Health and Wellbeing? A Cross-Sectional Online Survey
Source: Am J Lifestyle Med. 2026 Sep 21:15598276261484859. Online ahead of print. doi: 10.1177/15598276261484859 (PMC13593945; doi:10.1177/15598276261484859)
Supplement: Supplementary file 1 [file sj-zip-1-ajl-10.1177_15598276261484859.zip › Table Supplementary 4.docx]

**Table S4.** Paired comparison of prevention versus post-diagnosis acceptability (Q12 & Q13)

| **Statistic** | **Value** |
| --- | --- |
| N paired observations | 357 |
| Mean prevention score | 4.364 |
| Mean post-diagnosis score | 4.104 |
| Positive differences (prevention > diagnosis) | 104 |
| Negative differences (prevention < diagnosis) | 69 |
| Ties | 184 |
| Wilcoxon W (smaller rank sum) | 4745.5 |
| Two-sided p value | <0.001 |
