# Supplementary material for: Are Midlife Australians Ready to Implement Lifestyle Medicine to Improve Their Health and Wellbeing? A Cross-Sectional Online Survey
Source: Am J Lifestyle Med. 2026 Sep 21:15598276261484859. Online ahead of print. doi: 10.1177/15598276261484859 (PMC13593945; doi:10.1177/15598276261484859)
Supplement: Supplementary file 1 [file sj-zip-1-ajl-10.1177_15598276261484859.zip › Table Supplementary 4a.docx]

**Table S4a.** Distribution of paired score differences (prevention minus post-diagnosis)

| **Paired difference (prevention - diagnosis)** | **n** | **%** |
| --- | --- | --- |
| -2 | 8 | 2 |
| -1 | 61 | 17 |
| 0 | 184 | 52 |
| 1 | 57 | 16 |
| 2 | 34 | 10 |
| 3 | 7 | 2 |
| 4 | 6 | 2 |
