# Supplementary material for: Are Midlife Australians Ready to Implement Lifestyle Medicine to Improve Their Health and Wellbeing? A Cross-Sectional Online Survey
Source: Am J Lifestyle Med. 2026 Sep 21:15598276261484859. Online ahead of print. doi: 10.1177/15598276261484859 (PMC13593945; doi:10.1177/15598276261484859)
Supplement: Supplementary file 1 [file sj-zip-1-ajl-10.1177_15598276261484859.zip › Table Supplementary 5.docx]

**Table S5.** Seven-item scale calculation details

| **Component** | **Value** |
| --- | --- |
| Number of items (k) | 7.0 |
| Sum of item variances | 5.865546 |
| Variance of total summed score | 17.160104 |
| Cronbach alpha | 0.767885 |
| Overall mean (participant-level average) | 4.020008 |
| Overall SD (participant-level average) | 0.591782 |

**Table S5 (continued).** Item-level reliability statistics

| **No.** | **Scale item** | **Mean** | **Variance** | **Corrected item-total correlation** | **Alpha if item deleted** |
| --- | --- | --- | --- | --- | --- |
| Q12 | Interested in using LM for prevention | 4.364 | 0.513 | 0.565 | 0.73 |
| Q13 | Likely to use LM after new diagnosis | 4.104 | 1.009 | 0.304 | 0.78 |
| Q15 | Would like LM discussed at health check | 4.025 | 0.946 | 0.602 | 0.714 |
| Q17 | Personalised advice would encourage change | 4.23 | 0.554 | 0.63 | 0.717 |
| Q19 | Would like professional guidance/support | 3.81 | 1.009 | 0.591 | 0.716 |
| Q21 | Have family/friends support | 3.871 | 0.983 | 0.224 | 0.796 |
| Q22 | Ready to discuss lifestyle change with professional | 3.737 | 0.852 | 0.624 | 0.71 |
