# Supplementary material for: Are Midlife Australians Ready to Implement Lifestyle Medicine to Improve Their Health and Wellbeing? A Cross-Sectional Online Survey
Source: Am J Lifestyle Med. 2026 Sep 21:15598276261484859. Online ahead of print. doi: 10.1177/15598276261484859 (PMC13593945; doi:10.1177/15598276261484859)
Supplement: Supplementary file 1 [file sj-zip-1-ajl-10.1177_15598276261484859.zip › Table Supplementary 6.docx]

**Table S6.** Principal component analysis of the seven-item acceptability scale

| **Finding** | **Detail** |
| --- | --- |
| A dominant first component was observed | See eigenvalues in S9a and loadings in S9b |
