# Supplementary material for: Are Midlife Australians Ready to Implement Lifestyle Medicine to Improve Their Health and Wellbeing? A Cross-Sectional Online Survey
Source: Am J Lifestyle Med. 2026 Sep 21:15598276261484859. Online ahead of print. doi: 10.1177/15598276261484859 (PMC13593945; doi:10.1177/15598276261484859)
Supplement: Supplementary file 1 [file sj-zip-1-ajl-10.1177_15598276261484859.zip › Table Supplementary 6a.docx]

**Table S6a.** Eigenvalues and explained variance

| **Component** | **Eigenvalue** | **Proportion variance** | **Cumulative variance** |
| --- | --- | --- | --- |
| PC1 | 3.195 | 0.455 | 0.455 |
| PC2 | 0.981 | 0.14 | 0.595 |
| PC3 | 0.875 | 0.125 | 0.72 |
| PC4 | 0.619 | 0.088 | 0.808 |
| PC5 | 0.499 | 0.071 | 0.879 |
| PC6 | 0.465 | 0.066 | 0.945 |
| PC7 | 0.387 | 0.055 | 1.0 |
