# Supplementary material for: Are Midlife Australians Ready to Implement Lifestyle Medicine to Improve Their Health and Wellbeing? A Cross-Sectional Online Survey
Source: Am J Lifestyle Med. 2026 Sep 21:15598276261484859. Online ahead of print. doi: 10.1177/15598276261484859 (PMC13593945; doi:10.1177/15598276261484859)
Supplement: Supplementary file 1 [file sj-zip-1-ajl-10.1177_15598276261484859.zip › Table Supplementary 6b.docx]

**Table S6b.** Component loadings

| **No.** | **Scale item** | **PC1** | **PC2** | **PC3** | **PC4** | **PC5** | **PC6** | **PC7** |
| --- | --- | --- | --- | --- | --- | --- | --- | --- |
| Q12 | Interested in using LM for prevention | 0.405 | 0.037 | -0.026 | 0.803 | 0.093 | -0.241 | 0.35 |
| Q13 | Likely to use LM after new diagnosis | 0.245 | -0.404 | 0.836 | -0.137 | -0.088 | -0.224 | 0.003 |
| Q15 | Would like LM discussed at health check | 0.424 | -0.026 | -0.124 | -0.331 | 0.829 | -0.078 | -0.011 |
| Q17 | Personalised advice would encourage change | 0.439 | -0.199 | -0.004 | 0.207 | -0.087 | 0.723 | -0.441 |
| Q19 | Would like professional guidance/support | 0.429 | -0.133 | -0.26 | -0.399 | -0.4 | 0.149 | 0.624 |
| Q21 | Have family/friends support | 0.181 | 0.86 | 0.402 | -0.094 | -0.021 | 0.215 | 0.101 |
| Q22 | Ready to discuss lifestyle change with professional | 0.433 | 0.194 | -0.234 | -0.123 | -0.358 | -0.542 | -0.532 |
