# Supplementary material for: Are Midlife Australians Ready to Implement Lifestyle Medicine to Improve Their Health and Wellbeing? A Cross-Sectional Online Survey
Source: Am J Lifestyle Med. 2026 Sep 21:15598276261484859. Online ahead of print. doi: 10.1177/15598276261484859 (PMC13593945; doi:10.1177/15598276261484859)
Supplement: Supplementary file 1 [file sj-zip-1-ajl-10.1177_15598276261484859.zip › Table Supplementary 7.docx]

**Table S7.** Correlations between overall LM receptiveness and related constructs

| **No.** | **Predictor** | **Test** | **ρ** | **p value** | **N** |
| --- | --- | --- | --- | --- | --- |
| Q22 | Readiness to discuss lifestyle change | Spearman ρ | 0.743 | < .001 | 357 |
| Q8 | Learning about health is important | Spearman ρ | 0.349 | < .001 | 357 |
| Q10 | Motivated by LM learning/video | Spearman ρ | 0.514 | < .001 | 357 |
| Q2 | Awareness of LM term (0/1) | Spearman ρ | 0.102 | = .055 | 357 |
