# Supplementary material for: Are Midlife Australians Ready to Implement Lifestyle Medicine to Improve Their Health and Wellbeing? A Cross-Sectional Online Survey
Source: Am J Lifestyle Med. 2026 Sep 21:15598276261484859. Online ahead of print. doi: 10.1177/15598276261484859 (PMC13593945; doi:10.1177/15598276261484859)
Supplement: Supplementary file 1 [file sj-zip-1-ajl-10.1177_15598276261484859.zip › Table Supplementary 8.docx]

**Table S8.** Chi-square associations between readiness to discuss lifestyle change and key participant factors

| **No.** | **Factor** | **Chi-square** | **df** | **p value** | **Cramer's V** |
| --- | --- | --- | --- | --- | --- |
| Q32 | Chronic disease history | 7.647 | 2 | 0.0219 | 0.146 |
| Q2 | Awareness of LM | 2.925 | 1 | 0.0872 | 0.091 |
| Q25 | Gender | 0.083 | 1 | 0.7734 | 0.015 |
| Q26 | Age bracket | 9.335 | 4 | 0.0532 | 0.162 |
| Q31 | Education | 6.983 | 3 | 0.0724 | 0.14 |

| **No.** | **Related construct** | **Spearman rho** | **p value** |
| --- | --- | --- | --- |
| Q22 | Readiness to discuss lifestyle change | 0.743 | <0.001 |
| Q10 | Motivated to consider way of life | 0.514 | <0.001 |
| Q8 | Learning about health is important | 0.349 | <0.001 |
| Q2 | Awareness of LM term | 0.102 | 0.0547 |
