# Supplementary material for: Are Midlife Australians Ready to Implement Lifestyle Medicine to Improve Their Health and Wellbeing? A Cross-Sectional Online Survey
Source: Am J Lifestyle Med. 2026 Sep 21:15598276261484859. Online ahead of print. doi: 10.1177/15598276261484859 (PMC13593945; doi:10.1177/15598276261484859)
Supplement: Supplementary file 1 [file sj-zip-1-ajl-10.1177_15598276261484859.zip › Table Supplementary 8a.docx]

**Table S8a.** Chronic disease history, Awareness of LM, Gender, Age Bracket and Education: readiness cross-tabulation (counts and row percentages)

| **Factor** | **Positive n** | **Not positive n** | **Positive row %** | **Not positive row %** |
| --- | --- | --- | --- | --- |
| Chronic disease history: readiness cross-tabulation (counts and row percentages) | | | | |
| Yes | 118 | 56 | 68 | 32 |
| No | 96 | 74 | 56 | 44 |
| Not sure | 5 | 8 | 38 | 62 |
| Awareness of LM: readiness cross-tabulation (counts and row percentages) | | | | |
| Yes | 131 | 69 | 66 | 34 |
| No | 88 | 69 | 56 | 44 |
| Gender: readiness cross-tabulation (counts and row percentages) | | | | |
| Male | 47 | 32 | 60 | 40 |
| Female | 172 | 105 | 62 | 38 |
| Age bracket: readiness cross-tabulation (counts and row percentages) | | | | |
| 40-44 | 17 | 17 | 50 | 50 |
| 45-49 | 40 | 12 | 77 | 23 |
| 50-54 | 34 | 27 | 56 | 44 |
| 55-59 | 40 | 32 | 56 | 44 |
| 60-65 | 88 | 50 | 64 | 36 |
| Education: readiness cross-tabulation (counts and row percentages) | | | | |
| Did not finish school | 4 | 6 | 40 | 60 |
| High school | 38 | 29 | 57 | 43 |
| Undergrad | 78 | 33 | 70 | 30 |
| Postgrad | 97 | 70 | 58 | 42 |
