# Supplementary material for: Are Midlife Australians Ready to Implement Lifestyle Medicine to Improve Their Health and Wellbeing? A Cross-Sectional Online Survey
Source: Am J Lifestyle Med. 2026 Sep 21:15598276261484859. Online ahead of print. doi: 10.1177/15598276261484859 (PMC13593945; doi:10.1177/15598276261484859)
Supplement: Supplementary file 1 [file sj-zip-1-ajl-10.1177_15598276261484859.zip › Table Supplementary 9.docx]

**Table S9.** Acceptability means by chronic disease history (Q32)

| **Group** | **n** | **Mean acceptability** | **SD** |
| --- | --- | --- | --- |
| Chronic disease history: Yes | 174 | 4.096 | 0.557 |
| Chronic disease history: No | 170 | 3.954 | 0.616 |
