# Supplementary material for: Are Midlife Australians Ready to Implement Lifestyle Medicine to Improve Their Health and Wellbeing? A Cross-Sectional Online Survey
Source: Am J Lifestyle Med. 2026 Sep 21:15598276261484859. Online ahead of print. doi: 10.1177/15598276261484859 (PMC13593945; doi:10.1177/15598276261484859)
Supplement: Supplementary file 1 [file sj-zip-1-ajl-10.1177_15598276261484859.zip › Table Supplementary 9a.docx]

**Table S9a.** Independent-samples t test for acceptability by chronic disease history

| **Statistic** | **Value** |
| --- | --- |
| Welch t statistic | 2.245 |
| Welch degrees of freedom | 336.939 |
| p value | 0.0254 |
| Cohen's d | 0.242 |
