# Supplementary material for: Are Midlife Australians Ready to Implement Lifestyle Medicine to Improve Their Health and Wellbeing? A Cross-Sectional Online Survey
Source: Am J Lifestyle Med. 2026 Sep 21:15598276261484859. Online ahead of print. doi: 10.1177/15598276261484859 (PMC13593945; doi:10.1177/15598276261484859)
Supplement: Supplementary file 1 [file sj-zip-1-ajl-10.1177_15598276261484859.zip › Table Supplementary 9b.docx]

**Table S9b.** Acceptability means by age bracket

| **Age bracket** | **n** | **Mean acceptability** | **SD** |
| --- | --- | --- | --- |
| 40-44 | 34 | 3.815 | 0.769 |
| 45-49 | 52 | 4.231 | 0.486 |
| 50-54 | 61 | 4.026 | 0.579 |
| 55-59 | 72 | 4.077 | 0.493 |
| 60-65 | 138 | 3.959 | 0.612 |
