# Supplementary material for: Are Midlife Australians Ready to Implement Lifestyle Medicine to Improve Their Health and Wellbeing? A Cross-Sectional Online Survey
Source: Am J Lifestyle Med. 2026 Sep 21:15598276261484859. Online ahead of print. doi: 10.1177/15598276261484859 (PMC13593945; doi:10.1177/15598276261484859)
Supplement: Supplementary file 1 [file sj-zip-1-ajl-10.1177_15598276261484859.zip › Table Supplementary 9c.docx]

**Table S9c.** One-way ANOVA for acceptability by age bracket

| **Test** | **F statistic** | **df between** | **df within** | **p value** |
| --- | --- | --- | --- | --- |
| One-way ANOVA | 3.293 | 4 | 352 | 0.0114 |
