# Supplementary material for: Are Midlife Australians Ready to Implement Lifestyle Medicine to Improve Their Health and Wellbeing? A Cross-Sectional Online Survey
Source: Am J Lifestyle Med. 2026 Sep 21:15598276261484859. Online ahead of print. doi: 10.1177/15598276261484859 (PMC13593945; doi:10.1177/15598276261484859)
Supplement: Supplementary file 1 [file sj-zip-1-ajl-10.1177_15598276261484859.zip › Table Supplementary 9d.docx]

**Table S9d.** Tukey post hoc comparisons

| **group1** | **group2** | **meandiff** | **p-adj** | **lower** | **upper** | **reject** |
| --- | --- | --- | --- | --- | --- | --- |
| 40-44 | 45-49 | 0.4156 | 0.0119 | 0.0623 | 0.769 | True |
| 40-44 | 50-54 | 0.2106 | 0.445 | -0.1323 | 0.5535 | False |
| 40-44 | 55-59 | 0.2623 | 0.1987 | -0.0711 | 0.5956 | False |
| 40-44 | 60-65 | 0.1435 | 0.7022 | -0.1633 | 0.4502 | False |
| 45-49 | 50-54 | -0.205 | 0.3417 | -0.5074 | 0.0974 | False |
| 45-49 | 55-59 | -0.1534 | 0.6006 | -0.445 | 0.1382 | False |
| 45-49 | 60-65 | -0.2722 | 0.0358 | -0.5329 | -0.0115 | True |
| 50-54 | 55-59 | 0.0516 | 0.9866 | -0.2272 | 0.3304 | False |
| 50-54 | 60-65 | -0.0672 | 0.945 | -0.3135 | 0.1792 | False |
| 55-59 | 60-65 | -0.1188 | 0.6291 | -0.3517 | 0.1141 | False |
